# The invisible workforce during the COVID-19 pandemic: Family carers at the frontline

**DOI:** 10.12688/hrbopenres.13059.1

**Published:** 2020-05-15

**Authors:** Dominique Phillips, Gillian Paul, Majella Fahy, Linda Dowling-Hetherington, Thilo Kroll, Breda Moloney, Clare Duffy, Gerard Fealy, Attracta Lafferty

**Affiliations:** 1 UCD School of Nursing, Midwifery and Health Systems, University College Dublin, Dublin, Ireland; 2 UCD College of Business, University College Dublin, Dublin, Ireland; 3 Family Carers Ireland, Dublin, Ireland

**Keywords:** COVID-19, pandemic, family carers, employment, flexible working, policy

## Abstract

This is an open letter to acknowledge the essential and increasingly challenging role unpaid family carers are playing in the COVID-19 pandemic. The letter is written by members of the CAREWELL team, a HRB-funded project that aims to promote health and self-care behaviours among working family carers. Family carers provide care to family and friends in the community who need support due to old-age, disability and chronic illness. In many cases, family carers are supporting those who are considered most at risk in this pandemic meaning carers must reduce their own risk of infection in order to protect their dependent family members. The temporary reduction of some home care services, as well as school and creche closures, means that family carers are providing increased levels of care with little or no support. At a time when both worlds of work and care have been dramatically transformed, we wish to shed light on those who are currently balancing paid employment with a family caregiving role. We argue that there is much to be learned from the recent work restrictions that could benefit employees, including working family carers, beyond this pandemic. We also wish to build on the potential positives of a transformed society and encourage policy makers and employers to focus on what is currently being implemented, and to identify which measures could be used to create a bedrock of policies and practices that would offer robust and effective support to family carers. It is hoped that family carers will receive greater recognition for the significant role they play in society, providing essential care and alleviating the strain on health and social care systems, both during and post the COVID-19 pandemic.

## Disclaimer

The views expressed in this article are those of the authors. Publication in HRB Open Research does not imply endorsement by the Health Research Board of Ireland.

## Family carers and the COVID-19 pandemic

Although acknowledged as ‘key care partners’ ( Department of Health, 2012), family carers are often caring at the invisible level of health and social care systems and are continuing to be ‘unseen’ throughout the coronavirus disease 2019 (COVID-19) pandemic. Family carers provide essential support to people due to age-related issues, disability, addiction, mental health difficulties and chronic illness. These carers are alleviating pressure on increasingly over-stretched health and social care systems. In Ireland, it is estimated that between 4.1% and 10% of the population provide unpaid care ( CSO, 2016a; CSO, 2016b). Figures released by Care Alliance Ireland (2019) suggest that as many as 391,260 people in Ireland are providing care to a dependent relative. The value of informal care in Ireland has been estimated at between €2.1 and €5.5 billion per year ( Hanly & Sheerin, 2017), which gives some indication of the significant contribution carers make to society. The role of family carer continues 7 days a week, 365 days a year, regardless of public health and social measures such as school closures, restricted travel and business closures due to the national-health emergency.

As we now work together in a national response to control the transmission of COVID-19, this unpaid family care continues to be the backbone of care provision in the community. In many cases, people in receipt of this care are members of ‘high-risk groups’ ( Health Service Executive, 2020) and are at risk of developing serious illness if they contract COVID-19. As a result, carers are now providing even more support, while also under increased pressure to protect themselves from infection and prevent transmission to the person they care for. This presents many challenges particularly around providing personal care. Carers have had to adapt quickly as this pandemic has unfolded, often without clear guidance or personal protective equipment (PPE). Confinement that has been imposed on many, particularly on older people and those in need of support, may be very distressing, creating incredibly challenging home situations for some family carers. For example, some of the unique challenges faced by people with dementia and their carers have been recently highlighted ( O’Shea, 2020). These include the importance of protecting the personhood of people with dementia and offering stimulation and reminiscence activities and educational resources to help them and their carers during these difficult times. Others, such as individuals with an intellectual disability and those with autism may be finding changes in routine and activities particularly difficult, which in some cases may result in behaviours that can challenge ( College of Psychiatrists of Ireland, 2020). Also, the risk of abuse and domestic violence for family carers and those being cared for may increase as a result of the public health measures and restrictions ( Erosa *et al*., 2010). Consequently, physical distancing and isolation mean that this increase in violence and abuse may not be detected in a timely fashion.

Young carers, who are those under 18 with family care responsibilities, may be a particularly vulnerable group at this time. Attending school can be a form of respite where young carers can study for exams and focus on other aspects of their life ( Cluver *et al*., 2012). Due to school closures, young carers are now having to manage caring and schoolwork in the midst of managing their own and their loved one’s physical and mental health. There are other circumstances, for example, where family carers may not live with the person they are caring for, which raise issues in relation to travel, cross-contamination and decisions regarding the continued use of paid carers. Moreover, the cocooning of older family members may contribute to the acceleration of frailty and general deconditioning, which in turn, increases the need for support and care. The latter may place further strain on family caregivers, especially at a time of limited access to services. Family carers themselves may have underlying health conditions meaning that other family members, who may not have previously provided care, may now have to step in to fill a ‘care gap’. The suspension of many face-to-face services, respite care, school closures, and a reluctance to use some formal services that are available due to a fear of infection, means that family carers are now under tremendous strain, while providing more care than ever before and without the usual levels of support.

These circumstances are highlighted in survey findings recently released by Carers UK, which provide an insight into some of the new challenges, worries and concerns experienced by carers since the start of the pandemic ( Carers UK, 2020). Of the 5,047 carers who responded, 70% reported that they were providing more care due to the virus outbreak. Over a third of the participants were providing more care due to the closure of services and, on average, carers were providing an additional 10 hours care per week. Most of the surveyed carers were concerned about reaching a state of burnout in the coming weeks. Most notably, 87% of the carers in the survey were concerned about becoming ill and no longer being able to care for their loved one. Media coverage over the past few weeks suggests that family carers in Ireland are encountering the same kinds of challenges ( Irish Examiner, 2020; RTE, 2020a; RTE, 2020b; The Journal.ie, 2020). Family Carers Ireland have undertaken a similar survey of carers’ experiences during COVID-19 which findings should be published by the end of May. Family Carers Ireland are lobbying for family carers to be recognised as a vulnerable group and prioritised for COVID-19 testing and for PPE. They are also calling for the HSE to put an emergency contingency plan in place in the event of a carer or the person they care for contracting COVID-19. At this challenging time, there is an urgent need to prioritise the sustainability of current care arrangements by protecting the physical, psychological and financial well-being of carers.

## Considerations for working family carers

In Ireland, the majority of carers (54.6%) are in the paid labour force ( CSO, 2016a) and it has been estimated that as many as 1 in 9 employees were balancing work and care prior to the onset of this pandemic ( Family Carers Ireland, 2019). Factors such as an ageing population, more women participating in the workforce and longer working lives mean that we are likely to see an increase in the demand for care and those who balance work and care ( Oireachtas Library & Research Service, 2019). Under ‘normal’ circumstances and without adequate support, combining work and care can be challenging for many carers, and juggling these roles can impact their health and wellbeing ( Wang *et al*., 2011), and financial security ( Eurocarers, 2017). Many end up reducing their working hours, or leaving the workforce entirely, to provide care ( Dixley *et al*., 2019).

In response to the COVID-19 pandemic, the worlds of both employment and caregiving have been dramatically altered. Family carers are facing unique and unprecedented challenges in addition to the many pressures already associated with balancing work and family care. It is important to take cognisance of the considerable variation in the work-care situations that family carers may be facing. Many family carers are members of the ‘sandwich generation’ and are caring for both their own children and ageing parents. The challenge of managing these responsibilities alongside paid employment has been exacerbated by school and creche closures and the temporary withdrawal of other services such as respite and home care. Some family carers may be experiencing significant loss of income, and family carers who are considered ‘essential workers’ may feel conflicted and be concerned about leaving the home to go to work and risk infecting their loved ones. Family carers who may be employers or business owners may also be finding their multiple roles incompatible at this time of increased stress and uncertainty.

Research evidence suggests that enabling carers to remain in employment increases carers’ levels of happiness, financial security (including their future pension entitlements), and social inclusion ( Jungblut, 2015; Stiell *et al*., 2006). Furthermore, work can be a source of respite for many family carers as it provides time away from caring responsibilities ( Joseph & Joseph, 2019). However, as the lines between work and care become increasingly blurred, a growing number of carers may be at risk of burnout as many can no longer leave home to go to work. Also, longitudinal data suggests that without access to replacement care such as home care, personal assistant, day care, meals or respite, carers are more likely to leave employment entirely to fulfil their care role ( Pickard *et al*., 2018). Given the current suspension of so many of these key services, many carers may now be at an increased risk of employment exit. It is imperative that these individuals receive the appropriate services and financial support. Currently (7 ^th^ May 2020), carers who ‘voluntarily’ leave employment are not entitled to the Pandemic Unemployment Payment (PUP) (€350 weekly) (but may be entitled to apply for another welfare payment, paid at a lower rate than the PUP).

It is important to acknowledge that employers may not be aware of those who have caring responsibilities among their workforce. Research suggests that employees may be reluctant to identify themselves as carers due to perceived stigma or fear of negative career consequences ( Tehan & Thompson, 2013). This may be a source of great stress for some employees as they try to maintain the same workload with the additional strain of an increased ‘care load’. As this pandemic continues, employers and line managers, who have a responsibility to look after the wellbeing of employees, need to be understanding and find ways of sensitively opening discussions around care responsibilities and managing workloads.

Family carers themselves may be developing strategies such as making informal arrangements with line managers, taking parental leave, carers leave, sick leave or annual leave to help reconcile work and care. The state together with employers may need to consider how best to ensure that these employees are not unintentionally penalised, financially or otherwise, while providing this essential frontline service in the community. This seems reasonable considering the billions of Euros family carers save the state through the provision of unpaid care ( Hanly & Sheerin, 2017).

It is worth noting that recent work restrictions may not be an inherently negative experience for all. In fact, for some working family carers, there may be benefits associated with working from home, which may be enabling them to reconcile work and care more effectively. For example, working from home cuts down on commute times, allowing carers to spend more time with the person they care for. This applies particularly to carers who predominantly provide supervision and companionship to their relative, and whose assistance is required for just a few hours a day. Working and caring from home may also mean that care responsibilities can be more easily shared with other household members, helping to alleviate the pressure on the primary family carer. Indeed, there may be other positive consequences associated with caring during this stressful and challenging time. Benefit finding refers to positive psychological growth in times of adversity, illness or trauma and has been studied in relation to family caring ( Pakenham & Cox, 2008). Promoting or enhancing the identification of benefits such as a new appreciation of life, personal strength or improved relationships may help to improve the quality of life of carers post pandemic ( Brand *et al*., 2016).

Flexible working has been identified as one of the most effective strategies for assisting family carers in reconciling their caregiving and working responsibilities ( Eurocarers, 2017). However, working from home will not fully mitigate the increased strain that carers are under as they attempt to reconcile work and care during this pandemic. Without the appropriate supports, the provision of PPE equipment and the continuation and reinstatement of home care services, family carers are carrying an unacceptably heavy burden which is likely to have detrimental consequences for their physical, mental and financial wellbeing. Moreover, even when current restrictions are loosened and many people return to more familiar ways of working, some members of our community will continue to be at risk and will need to restrict their physical interactions with others for months to come. When non-essential workplaces begin to reopen, some groups of employees, such as family carers, may be reluctant to return to their place of work and risk carrying the virus home to their family. It is likely that COVID-19 will be a significant issue for working family carers for some time.

## What can we learn?

Despite the undisputed and varied challenges associated with this global health crisis, we also have a unique opportunity to maximise on learnings from the ways in which our home and work lives have been dramatically transformed. In the space of just a few weeks, remote working and working from home have become more normalised and ubiquitous than we could have ever previously imagined. Some employers have explored remote working for the first time and in many ways they have had to be more flexible than ever before. Therefore, the COVID-19 pandemic can offer us new insights, as many jobs which were previously not considered conducive to remote working have now had to rapidly become so.

As previously mentioned, offering such flexible working options has been identified as one of the most effective strategies to enable family carers to remain in employment while providing care ( Dixley *et al*., 2019). Learning from what has been successfully implemented over the past few months may help to accelerate the development of a national flexible working policy in Ireland. As part of Future Job Ireland, the Department of Justice and Equality conducted a public consultation in January 2020 on the development of this policy and sought the views of employees, employers, trade unions as well as the general public. The CAREWELL team made a submission to this consultation highlighting the importance of flexible working for employees with family care responsibilities ( Lafferty *et al*., 2020).

Similarly, lessons learned from COVID-19 may also help to inform the implementation of the EU’s Directive on Work-Life Balance for Parents and Carers. This Directive, which was adopted in June 2019, sets out minimum requirements related to parents and carers including the introduction of a minimum of 5 days of carers' leave each year for working family carers and the right to request flexible working arrangements for the purpose of providing care. Learning from the widespread implementation of flexible work arrangements may help to make workplace policies more accessible and responsive to employees’ needs in the future. If utilized effectively, the lessons from this global pandemic could help to foster carer-friendly workplaces and lead to more equitable work conditions that are likely to benefit all employees regardless of their care responsibilities.

While the full implications of the pandemic may take some time to determine, further research is warranted to ascertain the immediate and long-term impacts on family carers. For example, how will the cumulative burden from caregiving, economic strain, pressures on physical and mental health, and living with very stringent public health restrictions impact family carers’ resilience and ability to cope? Also, how will the pandemic impact young carers, their health and wellbeing, and their future career prospects? In particular, there is a need to understand the longitudinal impact, and the differential impact on working family carers. Are family carers more vulnerable to job losses as a consequence of the pandemic, and therefore have no choice but to become full-time carers to their loved ones? How will the pandemic affect certain groups of carers, such as the self-employed or female family carers in the labour market? Further research is required to ascertain the mitigating and positive factors emerging from the current situation in order to minimise potential inequalities and protect carers’ capacity to continue to provide essential care.

Throughout this pandemic, communities and families have worked together to provide key support to those who are most in need. As a society, we have pulled together to slow the spread of the virus and protect our most vulnerable citizens. As a consequence, previously invisible levels of health and social care delivery, including the contribution of family carers, may now become more widely acknowledged and recognised. By viewing this pandemic as an opportunity to inspire positive change, we may help to foster more caring workplaces, communities and society as a whole.

### The CAREWELL Project

The CAREWELL project is a four-year research project being undertaken by UCD, in partnership with Family Carers Ireland, to promote health and wellness among family carers in the workplace. The HRB-funded project also aims to examine strategies that enable family carers to combine caregiving responsibilities with work. For more information, please email CAREWELL@ucd.ie and visit the website www.carewellproject.com

## Data availability

### Underlying data

No data are associated with this article.
