# Supplementary material for: The invisible workforce during the COVID-19 pandemic: Family carers at the frontline
Source: HRB Open Res. 2020 May 15;3:24. [Version 1] doi: 10.12688/hrbopenres.13059.1 (PMC7276936; doi:10.12688/hrbopenres.13059.1)
Supplement: Peer review history

# Peer review: Reviewer response for version 1

**DOI:** 10.21956/hrbopenres.14155.r27417

**Published:** 2020-06-04

**Authors:** Maria Pierce

**Affiliations:** 1 Faculty of Science and Health, Dublin City University, Dublin, Ireland

As this Open Letter highlights, family carers have long been the backbone of care provision in the community, but often overlooked by government. Even before the outbreak of COVID-19, family carers often found themselves overstretched, due to fragmented, underdeveloped, and under-resourced home and community-based supports and services.

 This Open Letter is important and timely because of its focus on family carers who have remained largely invisible and relatively unsupported during the COVID-19 pandemic.

 The Open Letter pays particular attention to working family carers. It was written by the CAREWELL team, researchers at University College Dublin working in partnership with Family Carers Ireland to undertake a four-year research project to examine how family carers can best be supported to balance work with care. This provides the rationale for the focus on working family carers.

 The letter is not solely concerned with working family carers. The first part addresses the challenges for family carers generally during COVID-19. Since mid-February when the first public health advice was issued by government to ‘protect yourself’ against the COVID-19 by regular handwashing, good respiratory hygiene and not touching the face, family carers have been at the frontline of the national emergency. This Open Letter draws attention to the additional care load of, the increased pressures on and many challenges unfurled before family carers as a result of COVID-19. Insights from studies on the experiences of family carers of caring during COVID-19 and the profound impact that it is having are only beginning to emerge. The Open Letter refers to the survey undertaken by Family Carers Ireland examining the family carers’ experiences of caring in Ireland during the COVID-19 pandemic (which has since been published). The findings from this survey indicate the significant impact of caring through COVID-19 for family carers (Family Carers Ireland, 2020 ^1^) and reinforce the rationale of this Open Letter to make family caring more visible and to make a case for protecting the physical, emotional and financial wellbeing of family carers in order to sustain care arrangements.

 One point to be addressed in this first part of the letter is as follows. While it specifically highlights young carers as a particularly vulnerable group, there is no specific reference to older family carers. The only reference to older people is in relation to ‘cocooning’, and the potential to lead to the need for increased support and care by this group. While this is an important point, concerns have been raised that the discourse around older people during the COVID-19 crisis has been dominated by messages of frailty and vulnerability (e.g. O’Shea, 2020 ^2^). As data released by TILDA during COVID-19 has shown, 31% of over 70s provide help and care for their spouses, relatives (not including grandchildren), neighbours or friends (McGarrigle *et al*., 2020 ^3^). If this letter were to emphasise the significant contribution that older people make to society through their caregiving role, it could go some way to helping to rebalance this narrative and highlight the significant contribution that older people make to society in Ireland. Highlighting the contribution that older people make may also draw attention to the particular needs of and challenges facing older family carers, who have remained invisible throughout COVID-19, to the people they are providing care and support to, as well as other family members playing a secondary caregiving role who themselves may be working. 

 The second part of this letter focuses on working family carers (who we are informed make up more than half of all family carers, and as many 1 in 9 of all employees), the dramatic transformations in both formal work and informal caregiving as a result of COVID-19, and the unique challenges facing those combining working and caring. The Open Letter points to the evidence showing the benefits of enabling family carers to remain at work, and highlights the role and responsibilities of employers and the state in supporting working family carers and helping to moderate the additional stresses and strains that they are currently under. The Open Letter acknowledges the likelihood of increased employment exit for family carers as a result of measures such as the withdrawal of services, and the financial implications of this. Importantly, the letter stresses the considerable variations in work-care situations of family carers. As well as ‘voluntary’ exit from working, the authors may therefore wish to examine the drift into family caregiving during COVID-19 for some other workers who have lost jobs, either temporarily or permanently, or have experienced reduced working hours due to unprecedented closure of workplaces. As well as suddenly finding themselves in unchartered territory of family caring, returning to jobs or re-entering the labour market made be more complicated for workers who have taken on ‘new’ caregiving roles during the pandemic.

 The authors note the benefits associated with working from home for some working family carers, such as increased time availability as a result of shorter commuting times. It would be useful to note findings from research such as that from the European Foundation for the Improvement of Living and Working Conditions (2020) ^4^ which has shown that working from home does not always result in decreased working times, and for remote working to be sustainable, challenges such as overtime working need to be addressed, an issue that is especially pertinent for those combining work and caring. Time is really important to family carers, and in addition to considering how to make extra time available to family carers through workplace measures, it could be worth considering how certain issues around time could potentially be addressed through the reconfiguration of health and social care services (McDonald *et a*l., 2019) ^5^. Time taken up in this sphere has knock-on effects for working time. Technology may have a role to play in this regard.

 Given recent evidence of the heavily gendered nature of caring and unpaid work in Ireland (Russell *et al*., 2019 ^6^), the gendered impact of the COVID-19 outbreak (Wenham *et al*., 2020 ^7^) and concerns that COVID-19 is fuelling gender inequalities, it would also be interesting to consider working family caring though a gender lens, and encourage policy makers and employers to take a gender informed approach to policy and practice changes aimed at supporting working family carers. A work-related issue facing women predominantly, but not exclusively, that has been highlighted by the COVID-19 pandemic is the combining of formal and informal caregiving roles, referred to as double-duty or triple-duty caregiving. Research conducted in the US before the pandemic found that relative to their counterparts without family caregiving roles, women with combined formal and informal caregiving roles reported poorer psychosocial wellbeing (De Pasquale *et al*., 2016 ^8^), and it is likely that this has been exacerbated by COVID-19. Yet, jobs of workers with formal caregiving roles are unlikely to be amenable to remote working.

 This Open Letter on family carers caring through COVID-19 is to be welcomed. As COVID-19 restrictions are eased, the authors have shown that there are many lessons to be learned and research questions that remain to be answered. It ends on a hopeful note that as Ireland emerges from the pandemic the contribution of family carers will be acknowledged and recognised.

Does the article adequately reference differing views and opinions?

Partly

Are all factual statements correct, and are statements and arguments made adequately supported by citations?

Yes

Is the Open Letter written in accessible language?

Yes

Where applicable, are recommendations and next steps explained clearly for others to follow?

Yes

Is the rationale for the Open Letter provided in sufficient detail?

Yes

Reviewer Expertise:

Social policy, family care, health and social care, ageing and older people.

I confirm that I have read this submission and believe that I have an appropriate level of expertise to confirm that it is of an acceptable scientific standard.

## References

[1] Family Carers Ireland (2020) Caring Through COVID-19: Life in Lockdown. Reference source

[2] : Principles in the COVID-world.2020; Reference source

[3] : The Contributions of the Over 70s to Irish Society: Results from Wave 5 of the Irish Longitudinal Study on Ageing.2020; 10.38018/TildaRe.2020-01 10.38018/TildaRe.2020-01

[4] European Foundation for the Improvement of Living and Working Conditions (2020) Living, Working and COVID-19: First findings. *Publications Office of the European Union*. Reference source

[5] : 'Time is more important than anything else': Tensions of time in home care of older adults in Ireland. *International Journal of Care and Caring*.2019;3(4) :501-515

[6] : Caring and unpaid work in Ireland.2019; 10.26504/bkmnext382 10.26504/bkmnext382

[7] : COVID-19: the gendered impacts of the outbreak. *The Lancet*.2020;395(10227) : 10.1016/S0140-6736(20)30526-2 846-848 10.1016/S0140-6736(20)30526-2 PMC712462532151325

[8] : Combining Formal and Informal Caregiving Roles: The Psychosocial Implications of Double- and Triple-Duty Care. *J Gerontol B Psychol Sci Soc Sci*.2016;71(2) : 10.1093/geronb/gbu139 201-11 10.1093/geronb/gbu139 25271309PMC4757948

# Response

**Published:** 2020-06-15

**Authors:** Dominique Phillips

**Affiliations:** University College Dublin, Ireland

Dear Dr Maria Pierce,

On behalf of the CAREWELL team, I would like to thank you for your detailed, insightful and valuable feedback on our open letter. We will certainly take your comments on board in any future work relating to COVID-19 and the CAREWELL project and sincerely appreciate your time reviewing this article. We are currently conducting research with family carers whose employment has been impacted by COVID-19. It is hoped that this work will go some way in answering many of the points you have raised, particularly around balancing work and care.

Kind regards,

Dominique Phillips

# Peer review: Reviewer response for version 1

**DOI:** 10.21956/hrbopenres.14155.r27468

**Published:** 2020-06-03

**Authors:** Jo Aldridge

**Affiliations:** 1 Department of Social Sciences, Loughborough University, Loughborough, UK

This is a timely and important article/open letter that sets out clearly and concisely the evidence on informal (unpaid) care provision in families with a specific focus on caring during the COVID-19 pandemic. The authors are right to highlight the experiences and needs of family carers in the way that they have in this article and I am happy to fully endorse its indexing in HRB Open Research. The article is clearly expressed, is on an important topic with clear evidence to back up the claims and dialogue.

Does the article adequately reference differing views and opinions?

Yes

Are all factual statements correct, and are statements and arguments made adequately supported by citations?

Yes

Is the Open Letter written in accessible language?

Yes

Where applicable, are recommendations and next steps explained clearly for others to follow?

Yes

Is the rationale for the Open Letter provided in sufficient detail?

Yes

Reviewer Expertise:

Informal caring, young caring, vulnerable and marginalised people, participatory research and research ethics.

I confirm that I have read this submission and believe that I have an appropriate level of expertise to confirm that it is of an acceptable scientific standard.

# Response

**Published:** 2020-06-15

**Authors:** Dominique Phillips

**Affiliations:** University College Dublin, Ireland

Dear Professor Jo Aldridge,

On behalf of the CAREWELL team, I would like to thank you for taking the time to review our open letter and for your positive feedback and comments.  

Kind regards,

Dominique Phillips
